# Beyond the interface: benchmarking pediatric mobile health applications for monitoring child growth using the Mobile App Rating Scale

**DOI:** 10.3389/fdgth.2025.1621293

**Published:** 2025-06-18

**Authors:** Anggi Septia Irawan, Arie Dwi Alristina, Rizky Dzariyani Laili, Nuke Amalia, Arief Purnama Muharram, Adriana Viola Miranda, Bence Döbrössy, Edmond Girasek

**Affiliations:** ^1^ Institute of Behavioral Sciences, Semmelweis University, Budapest, Hungary; ^2^ Health Sciences Division, Doctoral College, Semmelweis University, Budapest, Hungary; ^3^ Sekolah Tinggi Ilmu Kesehatan Hang Tuah, Surabaya, Indonesia; ^4^ Safety and Health Engineering Study Program, Politeknik Perkapalan Negeri, Surabaya, Indonesia; ^5^ HealthAI Indonesia, Jakarta, Indonesia; ^6^ 1000 Days Fund, Denpasar, Indonesia

**Keywords:** pediatric care, stunting prevention, assessment, e-health, digital health, user experience (UX), user interface (UI)

## Abstract

**Introduction:**

As mHealth applications become increasingly adopted in Indonesia, it is crucial to assess their quality and usability for parents and healthcare professionals.

**Aim:**

This study evaluated the quality of pediatric-related mobile health (mHealth) applications available in Indonesia, focusing on their ability to support child growth monitoring and provide educational resources for parents and caregivers.

**Methodology:**

This is a cross-sectional study. From December 1, 2024, and January 31, 2025 we conducted systematic search for pediatric mHealth applications in Indonesian Google Play Store and Apple App Store using predetermined keywords. Inclusion criteria required the applications to be available in Bahasa Indonesia, focus on child health, and include growth tracking or stunting prevention features. We excluded applications that were not functioning during the testing period. Quality assessment was conducted by five healthcare professionals using the Mobile App Rating Scale (MARS). MARS assessed applications from multiple domains, including engagement, functionality, aesthetics, and information quality. Inter-rater reliability was ensured using the Intraclass Correlation Coefficient (ICC). The results were analyzed using descriptive statistics, Pearson's correlation, and T-tests. A *p*-value of <0.05 is considered to be statistically significant.

**Findings:**

Nine applications were included in this study. Seven of the applications (77.78%) focused on tracking child growth and development and providing educational content. Less than half of the apps had built-in community features that enabled social support (*n* = 4, 44.44%) and features for feedback mechanisms & personalized guidance (*n* = 3, 33.33%) respectively. The majority were developed by commercial companies (*n* = 7, 77.78%). Quality assessment found significant variability across the apps, with high functionality and aesthetics scores but more variability in the domains of app engagement, quality of information, and subjective quality or perceived value.

**Conclusion:**

This research underscored the need for the development of higher-quality, evidence-based mHealth apps for pediatric care in Indonesia, particularly in improving user engagement, feedback mechanisms and accessibility.

## 1 Introduction

The rapid advancement of mobile health (mHealth) technologies has significantly transformed healthcare delivery worldwide, particularly in pediatric care (1). MHealth applications offer innovative solutions for monitoring child growth, tracking developmental milestones, and providing health education to parents and caregivers (2). These digital tools have been shown to enhance parental knowledge (3), improve care seeking behavior (4), and support early childhood development interventions (4).

Smartphone penetration is projected to grow steadily between 2024 and 2029, with estimates suggesting it will reach 97 percent by 2029, an increase of over 15 percentage points (5). This consistent rise in smartphone adoption highlights the expanding potential of mobile platforms to support public health interventions (6). In this context, digital health solutions, particularly mobile applications, are increasingly recognized as valuable tools to facilitate early detection and intervention efforts, especially in areas such as child growth monitoring and early childhood development (7).

Studies suggested that mHealth apps can improve parental awareness, increase adherence to immunization schedules, and improve nutritional monitoring (8). Several pediatric-focused mHealth apps are available in Indonesia, including PrimaKu, Asianparent, and Tentang Anak, which offer features such as child growth monitoring, vaccination tracking, and parental education. However, the effectiveness of these applications depends on their usability, engagement, and the quality of the information they provide (9).

Assessing their quality using a standardized framework, such as the Mobile App Rating Scale (MARS), is essential to ensure they meet the needs of Indonesian parents and healthcare professionals (10). Despite the increasing adoption of mHealth applications, challenges remain in ensuring accessibility, credibility, and sustained user engagement (11). Many existing apps focus on tracking and information dissemination but lack of interactive features, such as feedback mechanisms, goal-setting, and social support, which are crucial for long-term user engagement (12). The user version of the MARS framework has been developed to incorporate user perspectives in the assessment process, which is essential for creating effective mHealth resources (13). Additionally, by integrating behavior change techniques into apps development can enhance alignment with users' desired health outcomes (14).

This study evaluated pediatric-related mHealth applications available in Indonesia, analyzing their quality using the MARS framework. By assessing key attributes such as engagement, functionality, aesthetics, and information quality, this research aimed to identify strengths and gaps in the current landscape of pediatric mHealth applications by MARS.

## 2 Methods

### 2.1 Study design

This study was designed as a cross-sectional analysis of mobile applications related to baby growth tracking, available in Indonesian App Stores. No regulatory approval was required for this study. The research was conducted under the Strengthening the Reporting of Observational studies in Epidemiology (STROBE) guidelines.

### 2.2 Raters selection

Five healthcare professionals were selected as raters (The data shown in Supplementary Table S1) based on the following criteria:

Inclusion criteria:

(i) Healthcare professionals or lecturers in the health sector, and/or (ii) Actively engaged in clinical practice in Indonesia.

Exclusion criteria:

(i) Not owning a mobile phone, (ii) Unable to download apps from the Apple App Store or Google Play Store, and (iii) Having hearing, visual, or motor impairments that could hinder participation.

### 2.3 Selection of the pediatric-related mHealth apps

Researchers selected mobile applications related to pediatric care and baby growth tracking between December 1, 2024, and January 31, 2025. The search was conducted in both the Indonesian App Store (iOS) and the Indonesian Google Play Store (Android). The keywords used for this search include: “pediatrik” (pediatric), “kesehatan bayi” (baby health), “stunting” (stunted growth), “pertumbuhan bayi” (baby growth), and “mengasuh anak” (parenting). Since the App Store and Google Play Store do not support the use of truncation or logical operators (AND, OR, NOT), each search term was entered separately.

To refine the selection, each researcher independently removed duplicate apps from the same app stores (iOS or Android). Subsequently, they compiled a unified list of apps available on both platforms to ensure accessibility for all users. After comparing their lists to verify completeness, the researchers downloaded the remaining apps to their devices and applied the inclusion criteria:

(1) The application must be available in Bahasa Indonesia,
(2) It must focus on pediatric care, and
(3) It must include at least one feature of baby growth tracking or stunting prevention features.

### 2.4 Evaluation of the pediatric-related mobile apps

#### 2.4.1 The use of standardized rating scale for mobile applications

This study utilized the original English version of the MARS for evaluation. The first component of MARS, known as “App Classification,” was assessed by two academic researchers. MARS is specifically designed to evaluate health-related mobile applications and consists of a primary section with 23 items divided into five categories (A, B, C, D, and E), along with an additional section (F) containing six items (The data shown in Supplementary Table S2). Each item on the MARS scale is rated on a 5-point Likert scale, A score of 1 indicates poor quality, while a score of 5 signifies high quality (10, 15).

#### 2.4.2 Evaluation of the apps by raters

The app evaluation was conducted by five academic health researchers. Before assessing pediatric care-related applications, the raters underwent training to familiarize themselves with the MARS. To ensure a consistent understanding of the MARS criteria, all raters participated in discussions to standardize their evaluation approach.

As part of the training, a test assessment was conducted using an app that was not included in the study sample. Each rater independently evaluated Halodoc, an app focused on general healthcare services rather than exclusively on pediatric care. The raters downloaded the app, tested its features for at least 15 min, and then completed the MARS assessment. Afterwards, they compared their scores. If any individual rating differed by 2 points or more, further discussion was held until a consensus was reached, ensuring uniformity in the evaluation process.

The formal assessment of pediatric care-related apps took place in February 2025. Each of the five raters downloaded and used each selected app for 15 min before completing the standardized online MARS questionnaire. During this evaluation, one application, Miki Anthropometri, was found to be unavailable and was subsequently excluded from the study.

### 2.5 Statistical methods

#### 2.5.1 Intraclass Correlation Coefficient (ICC)-raters

Ensuring the consistency of Mobile Application Rating Scale (MARS) scores across different raters, studies have employed the Intraclass Correlation Coefficient (ICC) as the primary method for assessing inter-rater reliability. The ICC is widely used for evaluating the reliability of measurements involving multiple assessors, particularly when working with ordinal or continuous data (16, 17).

A two-way random effects model with absolute agreement was chosen because it is specifically designed for situations where multiple independent raters provide evaluations, and the focus is on achieving absolute agreement rather than just relative consistency. This model effectively accounts for systematic differences among raters as well as measurement error, ensuring a more accurate assessment of reliability (17).

Since MARS scores are measured using a five-point Likert scale, the ICC serves as a robust indicator of variability across different evaluators while maintaining statistical precision. The reliability values were interpreted based on Cicchetti's classification (18). Guidelines, criteria, and rules of thumb for evaluating normed and standardized assessment instruments in psychology. In this study, ICC values ranged from 0.80 to 0.95, indicating a high level of agreement among the raters. These results confirmed strong inter-rater reliability, ensuring that the app evaluations were consistent and reproducible (19).

#### 2.5.2 Descriptive analysis

Descriptive analysis was used to describe the frequency distribution of app characteristics and research variables. The app characteristics are presented based on the theoretical background and strategy, affiliation, and technical aspects of the apps. The descriptive analysis in this study provided data on MARS mean scores and MARS scores for subcategories for each mobile health app, as well as boxplots to display the spread and distribution of scores.

#### 2.5.3 Statistics methods analysis

Pearson's correlation was used to analyze the correlation between apps and MARS variables (engagement, functionality, aesthetics, and information) with a *p* value of <0.05. An unpaired t-test was conducted to determine whether there was a statistically significant difference between the means of two independent groups, with a *p* value of <0.05. In this case, the total MARS mean scores of commercial and non-commercial apps were compared to assess whether commercial apps significantly outperformed non-commercial ones. The t-test helped determine whether the observed differences in mean scores were due to a genuine underlying effect or merely random variation.

#### 2.5.4 Heatmap visualization: method and justification

The patterns and variations in MARS were identified by scores across different applications and rating categories, a heatmap visualization was implemented (20). This method was selected for its ability to intuitively compare the relative performance of various applications across multiple dimensions, including engagement, functionality, aesthetics, information quality, and subjective quality.

The heatmap's color scheme followed a gradient from cool to warm tones, reflecting the magnitude of MARS scores. Cooler colors, such as blue and green, indicate lower scores, while warmer hues, like yellow, orange, and red, represent higher scores. Through this approach, key trends such as consistently high-performing apps, areas requiring improvement, and variations across different rating sections can be effectively identified.

Furthermore, the heatmap aided in recognizing consistency patterns across different evaluation criteria. For example, an application that scores consistently high across all categories will display predominantly warm tones, whereas one with mixed performance will exhibit a more varied color distribution.

## 3 Results

### 3.1 Selection of the pediatric care-related mobile apps

**Figure 1:**
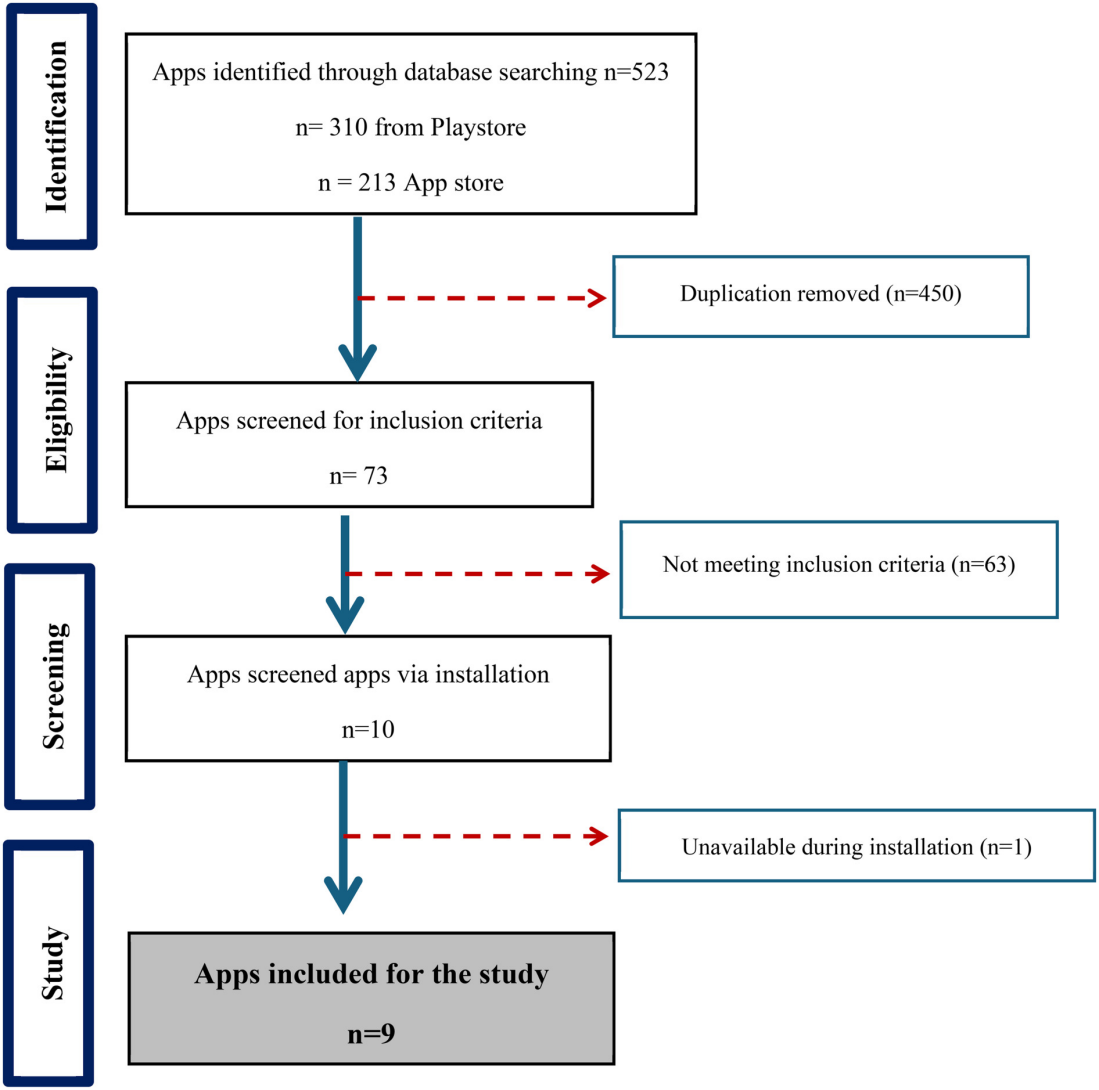
Flowchart of the pediatric care mobile apps selection.

A total of 13 apps in the App Store and 32 apps in the Google Play Store were identified (The data shown in Figure 1). The duplicates were eliminated in each list. The two lists were checked, analyzing the name of the app and the developer. 10 apps were available and selected on both systems. After downloading, one app was excluded because it was not functioning during the assessment. And only nine apps were included after screening.

### 3.2 General characteristics of the pediatric-related mobile apps

**Figure 2:**
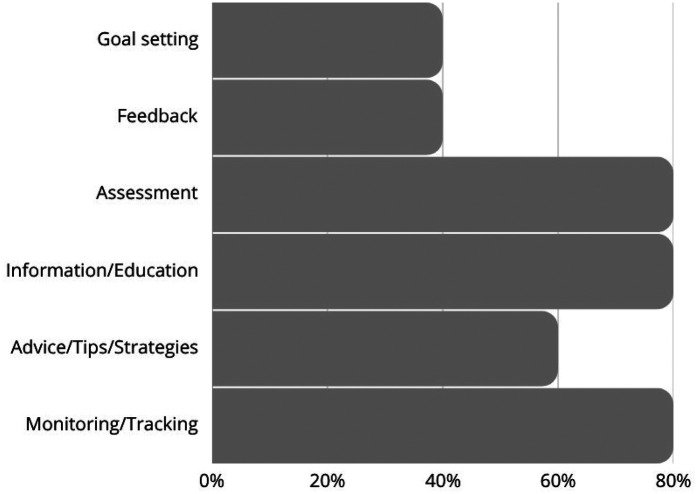
Apps characteristics based on theoretical background and strategies.

Among the nine analyzed mobile health applications (The data shown in Figure 1) for pediatric care, the majority (77.78%) incorporated monitoring and tracking features, highlighting their primary function as tools for continuous health data collection (The data shown in Supplementary Table S3). Similarly, from Figure 2 shown information and education components were present in 80% of the apps, indicating a strong emphasis on knowledge dissemination to caregivers and healthcare providers. Assessment capabilities were also observed in 80% of the apps, allowing for evaluations of child development and health status. In addition, Advice, tips, strategies, and skills training were also included in 60% of the apps, providing users with guidance on best practices for child health and nutrition. However, fewer apps included feedback mechanisms (40%) or goal-setting features (40%), suggesting limited interactive elements for user engagement and motivation.

**Figure 3:**
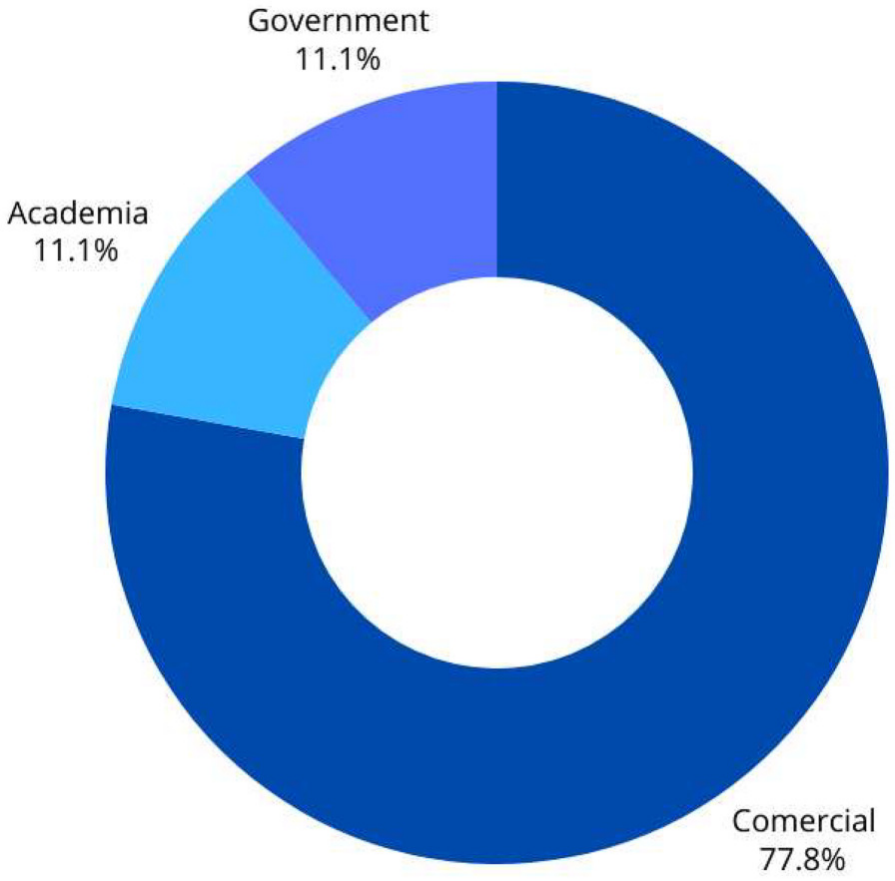
Apps characteristics based on affiliation.

Based on the affiliation (The data shown in Figure 3), most of the applications (77.78%) were commercially affiliated, while only one (11.11%) was developed by a government entity, and another (11.11%) was linked to the academic sector. The dominance of commercial applications suggested that the market for pediatric mobile health tools is largely driven by private sector initiatives rather than public health or academic research.

**Figure 4:**
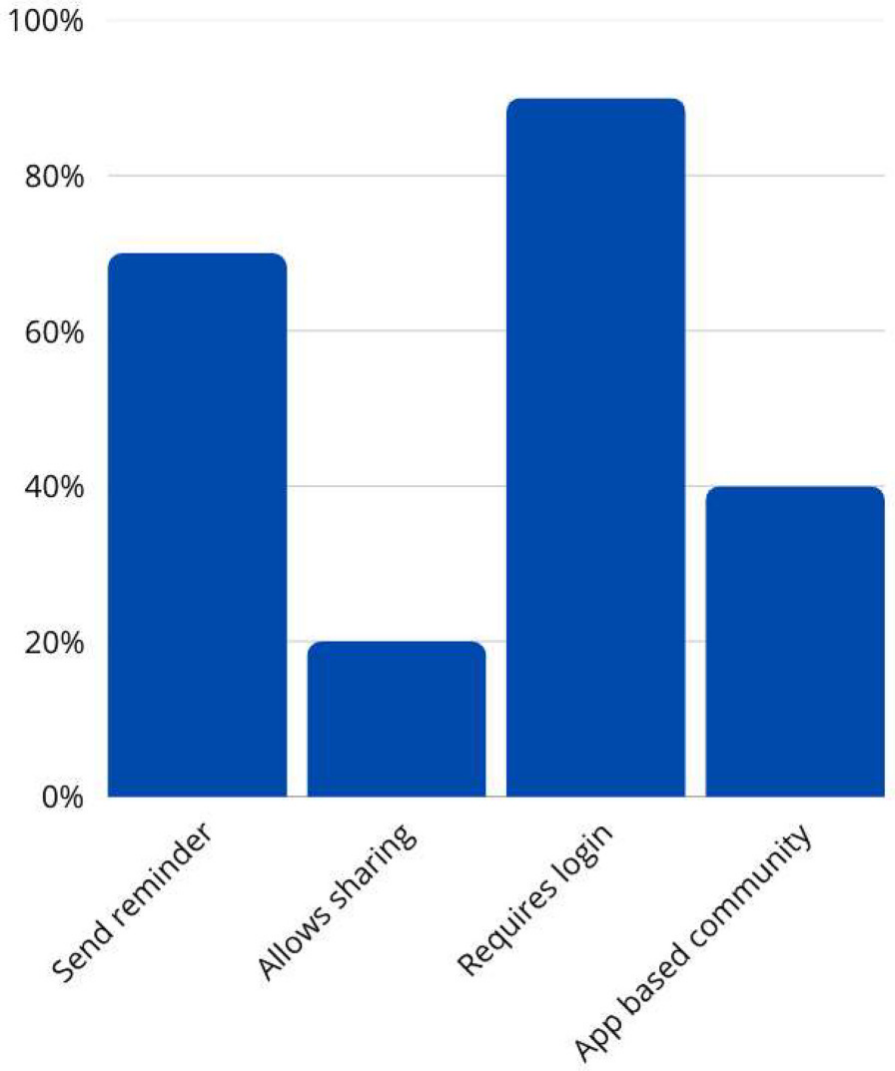
Apps characteristics based on technical aspects of the apps.

Regarding technical functionalities (The data shown in Figure 4), 70% of the apps provided reminder notifications, aiding users in maintaining consistent monitoring and engagement. However, only 20% allowed content sharing on social media platforms such as Facebook, which may limit peer support and community engagement. The majority (90%) required users to log in, possibly for data security and personalization purposes. Additionally, 40% featured an app-based community, which can enhance user engagement through peer interaction and support.

### 3.3 Comparison of MARS score

Four raters evaluated and rated the 9 apps analyzed (The data shown in Supplementary Table S4). The inter-rater agreement between the two raters was considered good, with Kendall's coefficient of concordance value of 0.93 and a *p*-value of 0.03.

**Figure 5:**
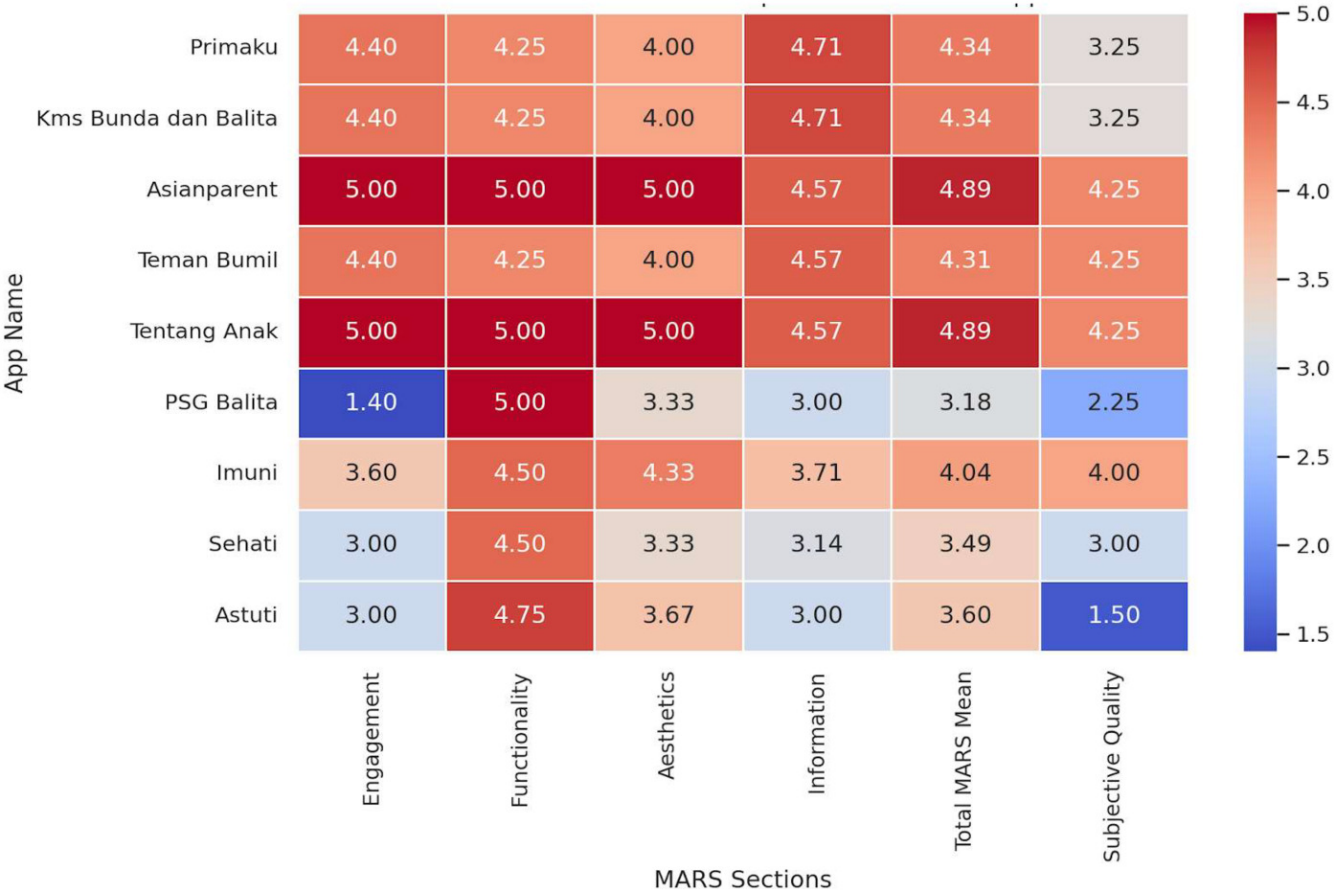
Heatmap visualization of comparison MARS mean score.

The heatmap visualization highlighted several key insights (The data shown in Figure 5). Among the high performers, Asianparent, and Tentang Anak stood out with consistently high scores across all sections. Their Engagement, Functionality, and Aesthetics scores were perfect (5.0), making them the most well-rounded apps. On the other hand, PSG Balita had a significantly low Engagement score of 1.40, despite achieving a high Functionality score of 5.00. In addition, Astuti had the lowest Subjective Quality score at 1.50, indicating notable shortcomings in user perception. Examining overall trends, *Functionality* appeared to be the most consistent category, with several apps scoring close to 5.0. However, *Engagement* scores varied widely, with some apps excelling while others struggling. Furthermore, *Information* scores were generally lower than other sections, suggesting a need for more credible and high-quality content.

The descriptive statistics of the MARS scores revealed notable trends across different evaluation sections. The *Engagement* section had a mean score of 3.80, but with a high standard deviation of 1.17, indicating significant variability among apps. Some applications, such as Asianparent and Tentang Anak, achieved perfect scores of 5.0, whereas PSG Balita scored the lowest at 1.40, reflecting a lack of interactive and engaging features. In contrast, the *Functionality* section demonstrated the highest consistency, with an average score of 4.61 and a low standard deviation of 0.33. This has shown that most of the apps provide a well-structured user interface with reliable performance and ease of navigation.

The Aesthetics scores exhibited moderate variability, with a mean of 4.07 and a standard deviation of 0.62. While apps like Asianparent and Tentang Anak scored 5.0, others, such as PSG Balita, scored notably lower at 3.33, indicating some inconsistency in visual design and stylistic appeal. The Information section had an average score of 3.99, slightly lower than the other categories, with a standard deviation of 0.78. These findings suggested that while some apps provide high-quality, evidence-based information, others might require improvements in content credibility and comprehensiveness.

The Total MARS Mean Score across all applications was 4.12, reflecting generally positive performance; however, individual app scores ranged from 3.18 to 4.89, indicating some variability in overall quality. Lastly, the Subjective Quality category, which represents users' perceived value of the apps, had the lowest mean score of 3.33, with the highest standard deviation of 0.98. These findings suggested a wide range of user experiences, with some apps being highly rated while others, such as Astuti with 1.50, facing significant user-perceived shortcomings.

**Figure 6:**
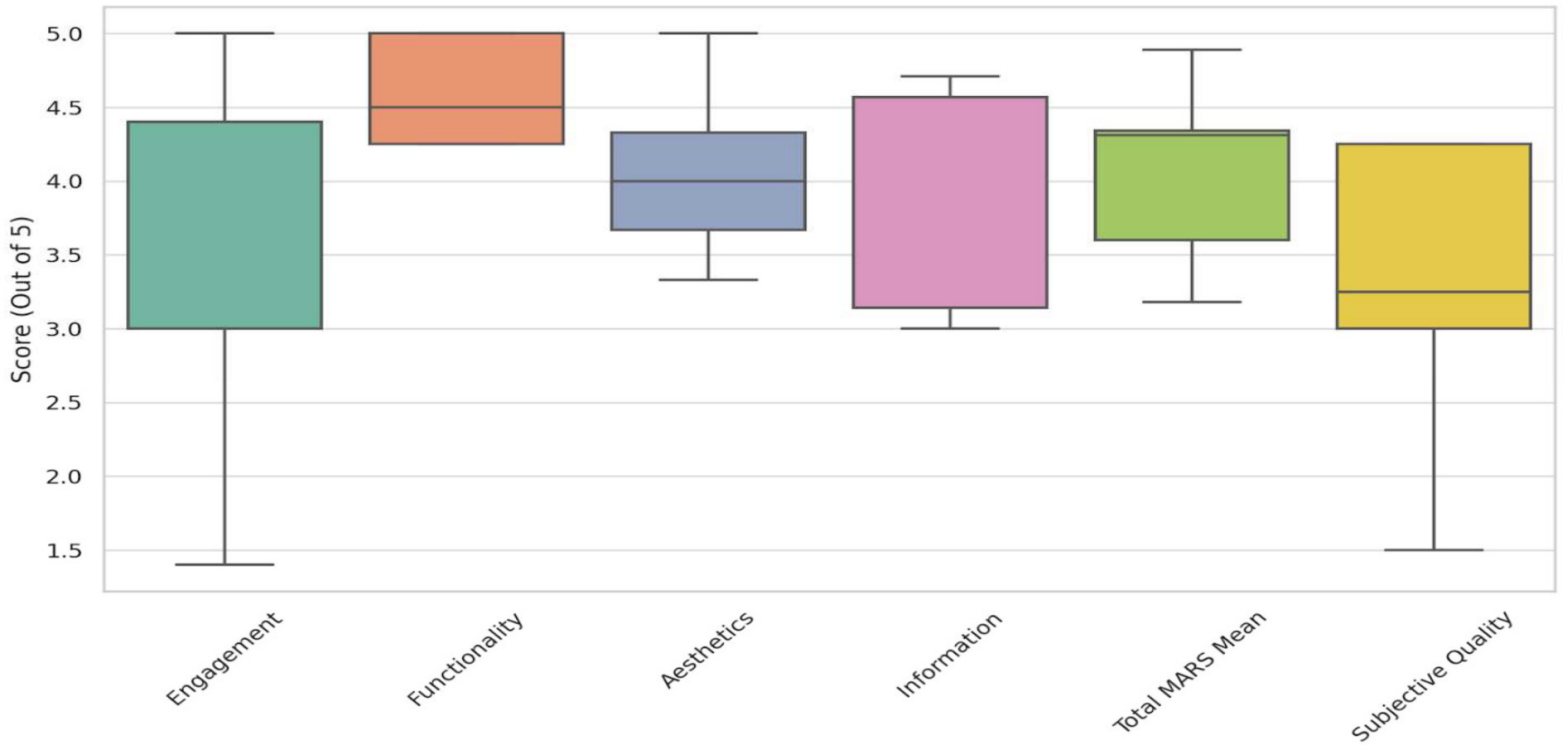
Boxplot of MARS scores across sections.

The boxplot (The data shown in Figure 6) visualization illustrated these variations, highlighting how *Functionality* remained consistently high across apps, while *Engagement* and *Subjective Quality* showed substantial fluctuations. The histogram distributions reinforced these findings, showing that while *Functionality* and *Aesthetics* tended to cluster around higher values, *Engagement* and *Subjective Quality* displayed more diverse patterns, suggesting areas where improvement is needed.

### 3.4 Correlation matrix MARS categories

**Figure 7:**
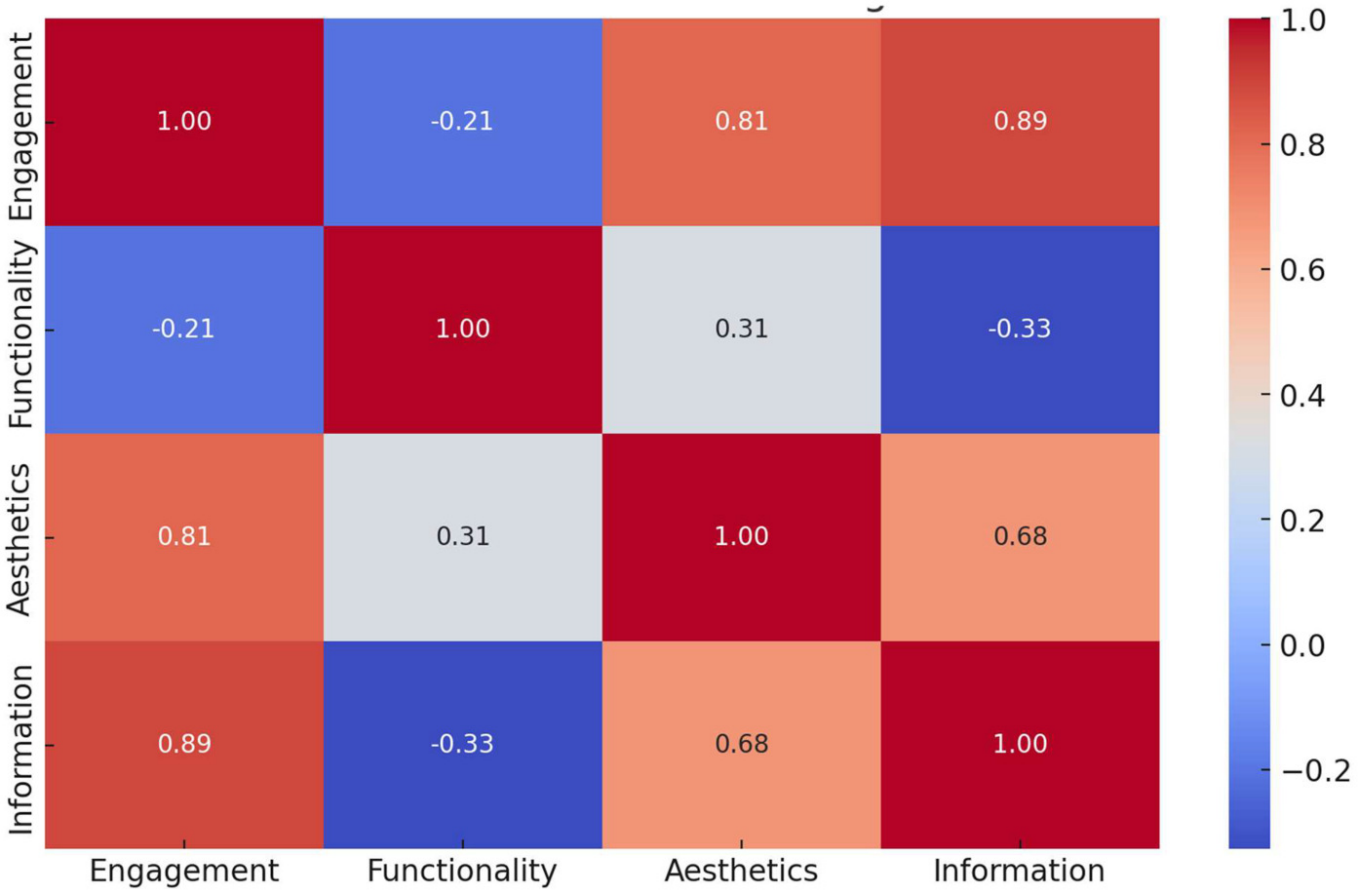
Correlation matrix MARS categories.

The statistical analysis revealed key insights into the variability and relationships among different MARS categories (The data shown in Figure 7). *Engagement* showed the highest variability, with a standard deviation of 1.17, indicating that user experience differed significantly across apps in terms of interactivity and appeal. In contrast, *Functionality* had the lowest variability (0.33), suggesting that most apps performed consistently well in usability and navigation. *Aesthetics* scores exhibited moderate variation (0.62), reflecting differences in visual design quality, while *Information Quality* also showed moderate variability (0.77), indicating that credibility and the depth of health information vary across apps.

The correlation analysis highlighted a strong positive correlation (0.89) between *Engagement* and *Information Quality*, suggesting that apps that successfully engage users also tend to provide high-quality and credible health information. However, the weakest correlation (−0.33) was observed between *Functionality* and *Information Quality*, indicating that a well-functioning app does not necessarily offer reliable medical content. These findings underscored the importance of balancing technical usability with medical accuracy, as strong functionality alone did not guarantee trustworthy health information. Developers should focus on enhancing both user experience and content credibility to ensure effective and reliable mobile health apps.

### 3.5 Behavioral outcomes result

**Figure 8:**
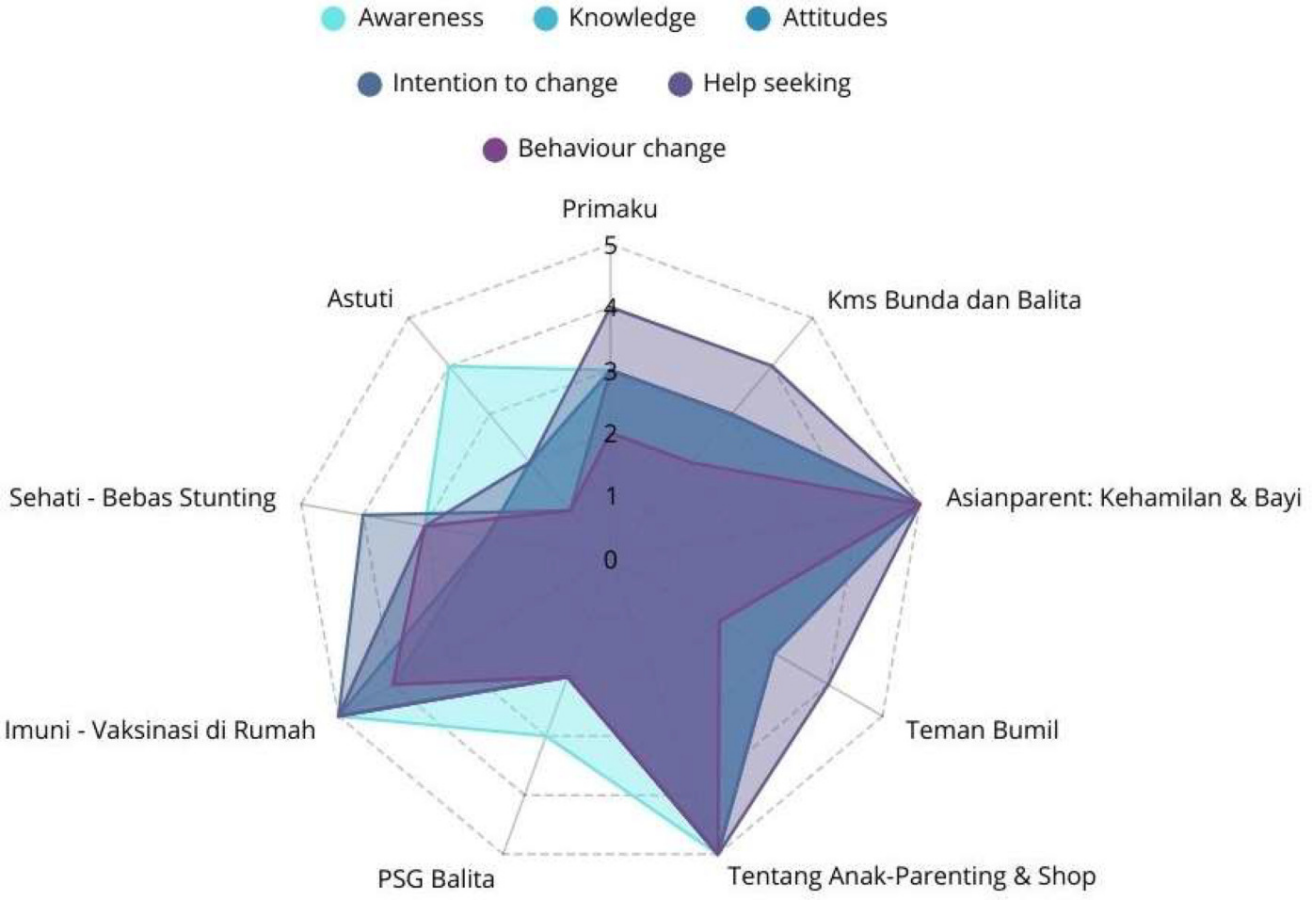
Average MARS behavioral outcomes by category.

Figure 8 showed the average score per behavioral category across all apps. Highest scoring categories included *Awareness* and *Help Seeking* (both averaging ∼3.78). Lowest scoring: *Behavior Change* (∼2.89), indicating that this was the area where most apps had the lowest scores (Data shown in Supplementary Table S5).

**Figure 9:**
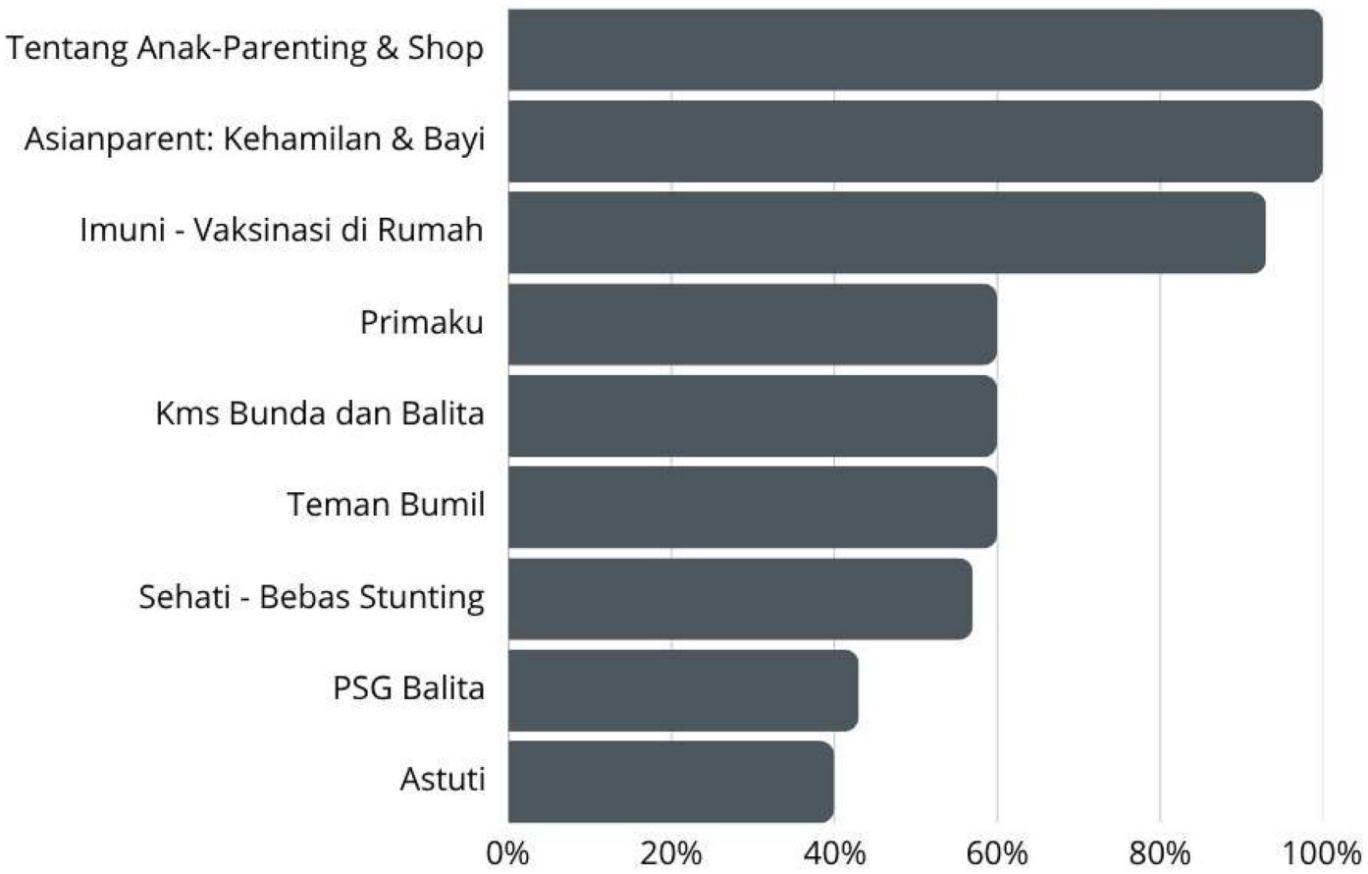
Average MARS behavioral scores by apps.

The analysis of behavioral outcomes among mobile health apps highlighted significant variations in their effectiveness. The Asianparent and Tentang Anak consistently achieved the highest scores across all categories (The data shown in Figure 9), demonstrating strong performance in raising awareness, enhancing knowledge, shaping positive attitudes, encouraging intention to change, promoting help-seeking behavior, and driving actual behavior change. Imuni, Primaku, KMS Bunda dan Balita, and Teman Bumil showed relatively strong results, particularly in help-seeking and behavior change, but did not reach the top-tier performance as Asianparent and Tentang Anak. On the other hand, PSG Balita and Astuti exhibited the weakest influence, with lower scores in key areas such as attitudes, intention to change, and behavior change, suggesting limited effectiveness in fostering health-related improvements. These findings indicated that while certain apps successfully drive user engagement and behavioral change, others might require enhancements in content, usability, and intervention strategies to maximize their impact.

### 3.6 Commercial vs. non-commercial apps

**Figure 10:**
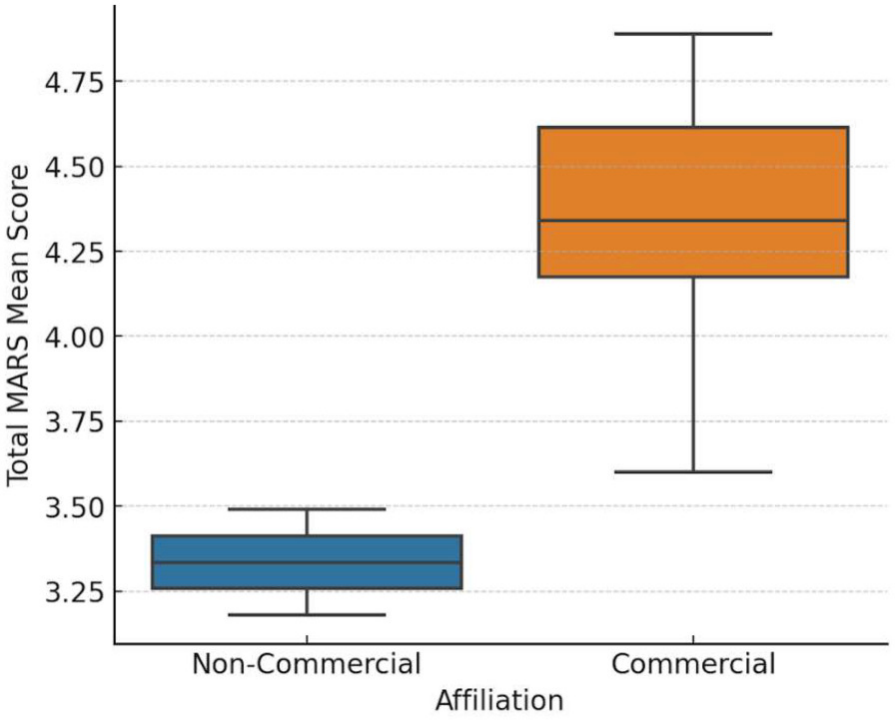
Comparison of MARS scores between commercial and non-commercial apps.

An independent t-test was conducted to compare the total MARS Mean scores between Commercial and Non-Commercial apps (The data shown in Figure 10). The results showed a statistically significant difference between the two groups, with a t-statistic of 4.36 and a *p*-value of 0.012 (*p* < 0.05), indicating that commercial apps tended to perform significantly better than non-commercial apps. Commercial apps had a significantly higher mean MARS score (4.34) compared to Non-Commercial apps (3.34). The *p*-value (<0.05) suggested that this difference is statistically significant, meaning the likelihood that this result occurred by chance is low. These findings suggested that commercial apps might have better resources, design, and engagement strategies, leading to higher overall quality.

## 4 Discussion

The comprehensive analysis provided insights into their functionality, design quality, engagement features, behavioral outcomes, and credibility of information. The findings demonstrated a wide variation in app quality, with clear patterns emerging between commercial and non-commercial apps. The analysis revealed that functionality remains a strong aspect across most apps, with a high mean score (4.61) and low variability. This indicated developers, particularly in commercial sectors, prioritize user-friendly interfaces, efficient navigation, and minimal technical glitches. This also aligned with previous research, highlighting usability as a critical factor in apps adoption and sustained usage (21–23).

However, usability alone does not equate to effectiveness in health communication or behavior change. One of the most striking findings was the high variability in engagement scores (mean = 3.80, SD = 1.17), which underscores disparities in how different apps maintain user interest. Apps such as Asianparent and Tentang Anak demonstrated strong engagement through features such as gamified content, push notifications, and community forums. This correlated with the findings by Stoyanov et al, which emphasized the significance of interactivity and tailored content in promoting continued use of health apps. On the opposite end, PSG Balita scored poorly, showing that technical performance without engaging elements was insufficient to keep users actively involved (10).

A closer look at information quality reveals another critical gap. Despite the high aesthetic and functional scores, several apps lacked well-sourced or expert-reviewed content. This confirms concerns raised by earlier studies that many commercially popular apps were not adhered to clinical guidelines or transparency regarding content sources (24, 25). The weak correlation (−0.33) between functionality and information quality in our data further underscored that a well-functioning app might still be inadequate in delivering trustworthy health information. This is concerning in the context of pediatric health, where misinformation can have serious consequences for child development and public health outcomes (26, 27).

Another important finding was the correlation between engagement and information quality (*r* = 0.89), suggesting the apps that were engaging also tended to present more credible information. This may be due to better resourcing or more holistic development strategies in commercially successful apps. It also supports theories from behavior change models, such as the COM-B framework (Capability, Opportunity, Motivation, and Behavior), which emphasized that both cognitive engagement and access to credible information are necessary to initiate and sustain behavior change (28, 29).

In terms of behavioral outcomes, apps performed moderately well in raising awareness and promoting help-seeking behavior but struggled with initiating long-term behavioral change. The average score for behavior change was the lowest among categories (mean = 2.89). This echoes similar findings by Zhao (30), who observed that many health apps fail to include evidence-based behavior change techniques (BCTs), such as goal setting, feedback loops, and rewards, that was critical for sustained impact. Given the burden of child malnutrition and stunting in Indonesia, the lack of BCTs significantly limited the apps' utility in public health interventions.

The comparative analysis between commercial and non-commercial apps revealed statistically significant differences in quality (*p* = 0.012), with commercial apps outperforming their counterparts in nearly all MARS categories. While this might be attributed to higher budgets, access to better design tools, and more aggressive user-testing, it raised ethical and equity concerns. Users from lower socioeconomic backgrounds might have limited access to premium features or might be exposed to advertisements and data privacy risks. This supported arguments that commercial models in child-focused mHealth might undermine public trust and dilute educational value (31, 32).

Our analysis indicated that applications with rich interactive features, including personalized dashboards, feedback systems, gamification, and real-time chat support, tend to receive higher user ratings and demonstrate stronger retention metrics. In contrast, applications lacking interactivity, such as those offering only static text or generalized health tips, are associated with lower engagement levels and higher dropout rates over time. This finding aligns with existing literature, which underscored the significance of user-centered design and engagement-driven features in sustaining digital health tool usage. Our benchmarking also showed that applications developed with active user input and designed around user-centered features are more likely to receive higher ratings and exhibit better retention metrics. This underscores the necessity for developers to prioritize not only the accuracy of health content but also the manner in which users interact with it. Incorporating engaging, responsive, and culturally appropriate features can improve the user experience and render digital tools more effective in supporting pediatric health outcomes. Conversely, applications that lack sufficient interactivity, such as those providing only static health information or basic notification systems, tend to experience lower user engagement. This is particularly evident in applications targeting parents in pediatric care, where usability and real-time feedback are essential. Low interactivity often results in reduced perceived value, limited user satisfaction, and ultimately, higher drop-off rates over time (33).

Existing studies revealed that, while mHealth applications share core features across different regions, their effectiveness is significantly influenced by local adaptation. Regional specificity in tool design, including the integration of local languages, is crucial, particularly in lower-middle-income countries, where numerous indigenous languages are spoken. For instance, a study conducted in Guangzhou, China revealed that 91.7% of the rural population was functionally illiterate and had not completed middle school, making it difficult for them to understand Mandarin or Cantonese (34).

This underscores the critical need for user-friendly interfaces and linguistic localization to enhance accessibility among the underserved populations. Moreover, in areas with limited Internet connectivity, such as parts of Sub-Saharan Africa, traditional communication methods, such as SMS and phone calls, are often preferred over internet-based applications. These platforms are more feasible for reaching users who lack smartphones or have stable Internet access. Therefore, to enhance the accessibility of mHealth in this group, two things need to be considered in designing and implementing the mHealth: the digital health literacy of the users and the supporting technological infrastructure (35).

Stronger collaboration between developers and policymakers is essential to address these challenges. Improving digital health literacy among decision makers can foster more effective engagement with technology developers and lead to more inclusive and sustainable solutions. Even when government staff possess technical expertise, low digital health literacy may hinder their ability to effectively evaluate and integrate new tools into health systems. Moreover, digital literacy skills in conjunction with the available supporting technological infrastructure can help in deciding which technology is appropriate for the users, such as SMS, phone calls, or mobile applications. Therefore, building capacity among public officials and ensuring their involvement in the design and scaling of digital health tools are critical steps toward improving accessibility for low-income and marginalized communities (34–36).

Despite providing valuable insights, this study had some limitations. First, the MARS evaluation, while comprehensive, remained a subjective assessment that might not fully capture long-term behavioral outcomes To gain a comprehensive understanding of the long-term impact of mobile health (mHealth) applications on pediatric health behaviors, future research should employ longitudinal study designs. Such methodologies facilitate an in-depth examination of behavioral changes over time, particularly concerning the persistence of user engagement and adherence to health recommendations. Long-term studies are especially critical for evaluating outcomes such as vaccination completion, breastfeeding duration, and compliance with growth monitoring schedules. Furthermore, it is imperative to investigate how user demographics, including age, socioeconomic status, education level, and digital health literacy, influence the engagement and overall effectiveness of mHealth tools. Previous research has indicated that disparities in digital health literacy, particularly among healthcare professionals, policymakers, and government personnel, can impede the optimal adoption and integration of these tools within national health systems (37). Furthermore, future research should focus more closely on technical design factors, such as the user interface (UI) and user experience (UX). Examining how diverse user groups interact with different app features, especially within large-scale public health programs, can provide valuable insights into developing more inclusive, accessible, and culturally sensitive digital platforms. Evaluating usability across various social, geographic, and infrastructural contexts will enhance the likelihood of adoption and improve long-term retention, which are essential for sustained health improvements. It is also important for future research to assess a larger and more diverse sample of applications, including mHealth tools from platforms beyond Bahasa Indonesia app stores, and monitor real-world usage over extended periods. These strategies will contribute to a more robust understanding of how mHealth applications perform across different populations and contexts, ultimately supporting the development of more effective, equitable, and user-centered digital health solutions for pediatric care in low- and middle-income countries.

## 5 Conclusions

This study applied the MARS framework to assess the quality and behavioral potential of nine pediatric mHealth apps in Indonesia. While many apps demonstrated strengths in functionality and aesthetics, significant gaps remained in engagement, information credibility, and behavioral impact. Commercial apps outperformed non-commercial ones, yet their dominance also raised concerns about equity and public interest.

To optimize the role of mHealth in reducing child stunting and improving pediatric care in Indonesia, future interventions must strike a balance between user-centered design and evidence-based health content. Multi-sectoral collaboration between developers, public health authorities, and academic institutions will be essential to develop inclusive, high-impact apps that not only inform but transform caregiver behavior and health outcomes.

## Data Availability

The original contributions presented in the study are included in the article/Supplementary Material, further inquiries can be directed to the corresponding author.
